# Development of the urogenital system is regulated via the 3′UTR of GDNF

**DOI:** 10.1038/s41598-019-40457-1

**Published:** 2019-03-28

**Authors:** Hao Li, Madis Jakobson, Roxana Ola, Yujuan Gui, Anmol Kumar, Petra Sipilä, Hannu Sariola, Satu Kuure, Jaan-Olle Andressoo

**Affiliations:** 1 Institute of Biotechnology, Helsinki Institute of Life Sciences and Faculty of Medicine, Helsinki, Finland; 2 Institute of Biomedicine, Biochemistry and Developmental Biology, University of Helsinki, Helsinki, Finland; 3 Present Address: Department of Basic, Preventive and Clinical Science, Faculty of Medicine, University of Transylvania, Brasov, Romania and Functional Genomics, Proteomics and Experimental Pathology Department, Prof. Dr. I. Chiricuta Oncology Institute, Cluj-Napoca, Romania; 4 Research Centre for Integrative Physiology and Pharmacology, and Turku Center for Disease Modeling (TCDM), Institute of Biomedicine, University of Turku, Turku, Finland; 5 GM-Unit, Laboratory Animal Centre, University of Helsinki, Helsinki, Finland; 6 Department of Neurobiology, Care Sciences and Society, Karolinska Institutet, Stockholm, Sweden

## Abstract

Mechanisms controlling ureter lenght and the position of the kidney are poorly understood. Glial cell-line derived neurotrophic factor (GDNF) induced RET signaling is critical for ureteric bud outgrowth, but the function of endogenous GDNF in further renal differentiation and urogenital system development remains discursive. Here we analyzed mice where 3′ untranslated region (UTR) of GDNF is replaced with sequence less responsive to microRNA-mediated regulation, leading to increased GDNF expression specifically in cells naturally transcribing *Gdnf*. We demonstrate that increased *Gdnf* leads to short ureters in kidneys located in an abnormally caudal position thus resembling human pelvic kidneys. High GDNF levels expand collecting ductal progenitors at the expense of ureteric trunk elongation and result in expanded tip and short trunk phenotype due to changes in cell cycle length and progenitor motility. MEK-inhibition rescues these defects suggesting that MAPK-activity mediates GDNF’s effects on progenitors. Moreover, *Gdnf*   ^hyper^ mice are infertile likely due to effects of excess GDNF on distal ureter remodeling. Our findings suggest that dysregulation of GDNF levels, for example via alterations in 3′UTR, may account for a subset of congenital anomalies of the kidney and urinary tract (CAKUT) and/or congenital infertility cases in humans and pave way to future studies.

## Introduction

Congenital anomalies of the kidney and urinary tract (CAKUT) are common birth defects affecting around 1% of live births, and causing most cases of the chronic kidney disease in children^1^. CAKUT covers a wide range of malformations that derive from deficiencies in embryonic kidney and lower urinary tract development, including obstruction of ureteropelvic junction, renal dysplasia, hydro-, ectopic and short ureters. Genetic causes for these malformations remain largely unknown despite the extensive sequencing efforts in gene coding regions^2^.

The morphogenesis of urogenital system is influenced by a common nominator, the nephric duct (ND), also known as Wolffian duct^3^. The ND extends caudally towards the posterior end of the embryo to connect to the cloaca through an endoderm-derived structure called the urogenital sinus. Proper positioning and timing of ND connection to the cloaca are important steps for the later development and function of the kidney and rest of the urogenital system. At least 1–2% of male infertility associates with ND defects^4^ and the development of Müllerian ducts, which give rise to oviducts (uterine tubes), the uterus and the upper part of vagina, depends on the normal caudal extension of ND^5^. An imperforate hymen is seen in 0.1% of newborn girls^6^ and 43% of vaginal and/or hymen agenesis patients co-present malformations in the kidney indicating common etiology between urinary and genital tract development^7^.

Development of the kidney begins by budding of the ND towards renal mesenchyme to create a ureteric bud (UB), which then undergoes extensive branching morphogenesis to generate the renal collecting duct system^8^. According to the “Ureteral Bud Theory” of Mackie and Stephens (1975) and supported by recent transgenic studies^9^, the site where the UB forms greatly influences ureter maturation and determines the final position of the connection to the bladder. Specifically, cranial budding results in failure to segregate the ureter from the ND, resulting in obstruction, while caudal budding results in the ureter connection to more lateral or anterior sites on bladder, leading to reflux^10^.

Concomitantly with UB branching begins the distal ureter maturation, which remodels the most caudal segment of ND, also called the common ND. The vertical displacement process moves the ureter upwards and separates it from the ND, which in males differentiates into the *vas deferens* and epididymal ducts, and in females plays an important function in Müllerian duct guidance^9,11,12^. The urogenital sinus is an important signaling center expressing several hormones and receptor tyrosine kinase ligands such as GDNF^13,14^ that may influence ND to cloaca attachment and distal ureter remodeling. The regulation of distal ureter remodeling is beginning to emerge^15^, but how ureter length and anatomical positioning of urogenital organs occur are less well understood.

It is well established that kidney development critically depends on GDNF induced RET tyrosine kinase signaling^16^ (see also Table 1). Mice lacking *Gdnf*, receptor *Ret* or co-receptor *Gfra1* show renal aplasia while those with specific mutations in *Ret* have additional impairment in ND differentiation^12,17–19^ and display phenotypes that are not reported in *Gdnf* knockouts^20–22^. Ectopic transgenic misexpression of *Gdnf* in ND and derivatives suggested that GDNF is not chemoattractive signal for ureteric bud but stimulates excessive ectopic budding and frequent defects in ureter to bladder connection^23^. Similar excess budding is seen in mouse models lacking SLIT2-ROBO2 signaling which is required to restrict *Gdnf* expression to its normal domain, and *Sprouty1*, a protein needed to negatively regulate activation of receptor tyrosine kinase signaling^24,25^. These results suggest that normal nephrogenesis necessitates critically regulated *Gdnf*

**Table 1:** Summary of GDNF functions identified using GDNF^hyper^ mice.

Published overexpression/functional inactivation studies		Endogenous GDNF elevation (this study)	
*Phenotype*	*Strategy*	*Phenotype*	*Novelty*
ND: extra budding rostrally	Exogenous GDNF protein in kidney explant cultures^57^	ND: normal until common ND remodelling	New
UB formation: not affected		UB formation: abnormally wide UB	New
Kidney proper: expanded UB tips		Kidney proper: expanded UB tips	In-line
ND: extra budding along the entire ND & ureter connecting to sex ducts	Inducible, ectopic GDNF over-expression in ND^23^	ND: normal until common ND remodelling,	New
		& ureter connecting to sex ducts	In-line
Mesonephros: extra budding		Mesonephros: normal	New
Kidney proper: expanded UB tips		Kidney proper: expanded UB tips	In-line
ND: normal	Conventional *Gdnf* deletion^20–22^	ND: normal until common ND remodelling	In-line
UB formation: not affected		UB formation: abnormally wide	New
Kidney proper: no kidney		Kidney proper: short UB trunk & pelvic kidney	New
ND: extra budding rostrally	Conventional deletion of Robo2/Slit2 (needed to restrict *Gdnf* expression)^24,58^	*ND*: normal until common ND remodelling	New
UB formation: not reported		UB formation: abnormally wide UB	New
Kidney proper: expanded UB tips, several ureters, hydroureters		Kidney proper: expanded UB tips, occational hydroureters	New & In-line
ND: extra budding rostrally	Conventional deletion of Spry1 (negative inhibitor of Ret signaling)^24,25,59,60^	ND: normal until common ND remodelling	New
UB formation: abnormally wide UB		UB formation: abnormally wide UB	In-line
Kidney proper: expanded UB tips, several ureters, hydroureters, blind ended ureters		Kidney proper: expanded UB tips, occational hydroureters	In-line
RPO: ureter connecting to vas deference, abnormally located testes		RPO: Failure to separate ureter	New
		from common ND, vaginal imperforation, abnormal connection between vas deference and seminal vesicle, sperm in	New & In-line
		seminal vesicle, infertility	New
RPO: Proliferative response & increase in urethral mesenchyme thickness	Exogenous GDNF protein in urogenital sinus explant cultures^45^	RPO: Failure to separate ureter from common ND, ureter and sex duct misconnections	New
RPO: Increase in ovarian primordial follicle development	Exogenouse GDNF protein in ovary explant cultures^61^	RPO: Vaginal imperforation, infertility	New
RPO: Patchy loss of spermatogenesis -> normal fertility	GDNF haploinsufficiency, *Gdnf*+/−^43^	RPO: Sperm in the of seminal vesicle, abnormal connection between vas deference and seminal vesicle,	New
RPO: Spermatogonial differentiation failure, accumulation of stem cells -> infertility	Ectopic GDNF overexpression with EF1 promoter^43^	infertility without spematogonial differentiation defect	New & In-line

The main findings of this study are reported in comparison with published reports from ectopic GDNF applications and GDNF knock-out mice. The forth column indicates whether the observations made in GDNFhyper mice are consistent with function predicted from ectopic GDNF applications and from Gdnf gene deletion studies or provide novel information. Abbreviations: ND; nephric duct, RPO; reproductive organ, UB; ureteric bud.

expression. However, what other functions endogenous GDNF has in developing kidney e.g. in regulating ureteric bud morphogenesis and differentiation, or differentiation of ND associated reproductive organs remains to be studied.

Previously, we showed that microRNAs miR-9, miR-96, miR-133b and miR-146a suppress GDNF expression by interaction with *Gdnf* 3′UTR, and that *in vivo* replacement of *Gdnf* 3′UTR with a sequence less responsive to regulation by microRNAs and RNA-binding proteins leads to increased endogenous GDNF expression^26^ (*Gdnf*^*hyper*^). Our previous analysis focused on brain dopamine system development and function^26^, mainly because of high hopes for using GDNF in Parkinson’s disease treatment and ongoing clinical trials. We also reported that quite unexpectedly for a kidney morphogen, *Gdnf*^    *hyper/hyper*^ mice have small and malformed kidneys. Importantly, endogenous *Gdnf* expression pattern is maintained in *Gdnf*^    *hyper/hyper*^ mice, which have kidneys and are rescued from the early postnatal lethality caused by *Gdnf* deletion^26^. This allows examination of GDNF functions beyond ureteric bud induction and in postnatal pups.

Here we found that GDNF regulates ureteric bud trunk length through the control of collecting ductal progenitors and by this way defines the position of kidneys in the abdominal cavity. GDNF coding region aberrations are rare in CAKUT patients and occur mostly together with mutations in RET (Table 2). However, our results illustrate the pathogenic potential of dysregulation in GDNF levels and suggest that such analysis, though technically challenging in patients, may reveal new pathogenesis of CAKUT.

**Table 2:** Overview of mutational analysis in GDNF/RET signaling components in patients with congenital anomalies of kidney and urinary track.

Publications analyzing GDNF/RET/	Identified variantion			Cohort & association with kidney	*Notes*
GFRa1 aberrations in humans	*RET*	*GDNF*	*GFRa1*		
Skinner *et al*.^63^	yes	***only concurrently with RET variant (1)***	no	33 stillborn fetuses/aplasia or severe dysplasia	A *GDNF* variant detected in only one fetus with unilateral agenesis and additional mutation in *RET*
Yang *et al*.^64^	yes	*NA*	NA	118 Canadian pVUR patients	70% of the patients carry SNP in *RET* potential phosphorylation site
Zhang *et al*.^62^	yes	*NA*	NA	136 full-term healthy infants	A common *RET* variant associates with reduced renal size & function
Jeanpierre *et al*.^53^	yes	**NO**	NA	105 fetuses with bilateral renal defects (agenesis, hypodysplasia, multicystic dysplasia)	Analyzed coding, promoter & 3′UTR regions + copy number variations to identify low frequency of potential *RET* mutations
Chatterjee *et al*.^52^	yes	**yes (2)+ concurrent-** ***ly with RET variant (1)***	yes (1)	122 unrelated CAKUT patients	A *GDNF* variant detected concurrently with RET variant in one VUR, and alone in one VUR+ ectopia&hydronephro− tis and one VUR + unilateral agenesis&ectopia patient
Kaczmarczyk *et al*.^65^	yes	NA	no	188 full-term healthy infants	Confirms the association of common RET variant with reduced renal size & function reported by Zhang *et al*.^62^

Targeted and whole genome sequencing approaches have revealed genetic aberrations in GDNF receptor *Ret* but mutations in *Gdnf* itself are largely either missing or in combination with *Ret* variations. Abbreviations: NA; not analyzed, pVUR; primary vesicoureteral reflux, VUR; vesicoureteral reflux.

## Results

### Genetically enhanced endogenous GDNF expression shifts expansion of primary ureteric bud rostrally and distorts ureter morphogenesis

**Figure 1:**
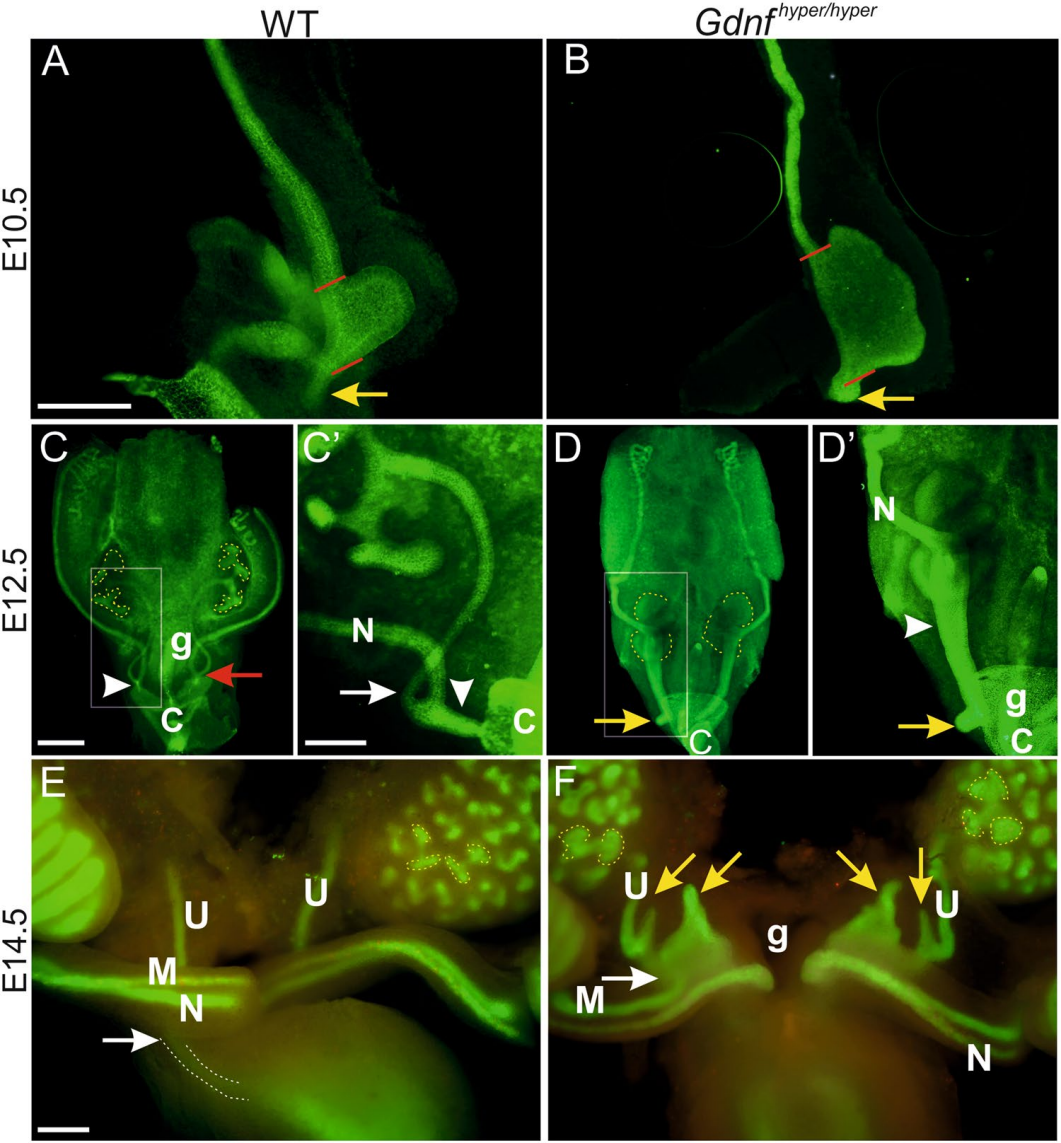
Ureteric bud and distal ureter morphogenesis are severely disturbed in embryos with enhanced GDNF levels. Whole-mount calbindin staining of E10.5 (**A**) wild type (WT) and (**B**) *Gdnf* ^hyper/hyper^ urogenital blocks. Red lines show the width of the primary bud, yellow arrow points to normal (WT) and expanded (*Gdnf* ^hyper/hyper^) end of nephric duct. Whole mount E-cadherin staining of E12.5 (**C**) WT and (**D**) *Gdnf* ^hyper/hyper^ urogenital blocks. Ureteric bud tips are depicted by yellow, dotted lines, arrowheads point to common nephric duct, which failed to start remodeling in *Gdnf* ^hyper/hyper^ urogenital system, white arrow shows the distinction of ureter from nephric duct in WT control (**C’**). Red arrow in C indicates the side where vertical displacement has completely occurred, yellow arrows show extra ureteric budding near to cloaca in the posterior nephric duct (**D**, **D’**). (**E**) E14.5 WT sample stained with E-cadherin (green) and cleaved-Caspase3 (red) shows normal ureteric buds (yellow, dotted lines) and ureters connecting to the lateral sides of upper bladder (white dotted line pointed by white arrow), while (**F**) in *Gdnf* ^hyper/hyper^ embryos ureters show large extension-like extra buds (yellow arrows) that connect to sex ducts (white arrow) and dilatated ureteric bud tips (yellow, dotted lines). Abbreviations: C; cloaca, g; gut, M; mesonephric duct, N; nephric duct, U; ureter. Scale bars: 200 µm.

We have previously reported that GDNF protein levels are increased 3-fold in *Gdnf*  ^*wt/hyper*^ and 6-fold in homozygous kidneys of *Gdnf *^   *hyper/hyper*^ mice at embryonic day 18.5 (E18.5) and result in reduced kidney size at birth^26^. Here we investigated how GDNF 3′UTR regulation, which does not affect endogenous expression pattern, influences the development of the whole urogenital system, where excess *Gdnf* expression is confined in the cells endogenously expressing it (Fig. S1A–E). We first analyzed nephric duct (ND) morphogenesis and UB morphology at early developmental stages. After its formation, the ND extends caudally towards the posterior end of the embryo to connect to the cloaca (Figs. 1A, S1E,F). At E10.5, the ND was connected to the cloaca in both control and *Gdnf*   ^hyper/hyper^ embryos but the connecting segment of ND itself was dilated in the *Gdnf *^hyper/hyper^ embryos (Fig. 1A,B). As previously described, the UBs were also wider in *Gdnf*  ^hyper/hyper^ than those in wild type (WT) embryos (Fig. 1A,B). Moreover, the primary bud in *Gdnf*  ^hyper/hyper^ embryos was formed more rostrally than in the WT embryos, placing the ureteric budding site to an abnormal location. According to the budding theory, this would predict ureter obstruction during later development^10^.

We next studied further nephric duct morphogenesis and UB branching at specific developmental stages known to be critical for ND remodeling, distal ureter remodeling and ureter maturation^9^. At the onset of UB branching morphogenesis, distal ND showed distinct segment of connecting piece and separation of ureter from ND in WT embryos, while *Gdnf*  ^hyper/hyper^ and fraction of *Gdnf*  ^wt/hyper^ embryos (45%, unilateral) showed abnormal UB morphology and failure to segregate distal ureter from ND (Fig. S2A–C). After branching morphogenesis initiation, WT kidneys typically showed several UB tips (8–9/kidney at E12.5) (Fig. 1C). Vertical displacement was either initiated (left side) or completed (right side) in the WT at E12.5 (Fig. 1C-C’). At this point, ureteric branching was severely retarded in the *Gdnf*  ^hyper/hyper^ embryos (Fig. 1D). Also extra budding was seen at the rostral end of the *Gdnf*  ^hyper/hyper^ common ND, which failed to start normal vertical displacement and exhibited a dilated common ND (Fig. 1D-D’). The common ND remodelling was completed in WT embryos by E13.5 as seen by separated ureter and nephric duct (Fig. S2D). The common ND in *Gdnf*  ^hyper/hyper^ embryos though remained aberrantly long and showed a widely dilated caudal end (Fig. S2E). Similar ND abnormalities were still present in *Gdnf*  ^hyper/hyper^ embryos at E14.5 and interfered with Müllerian duct development as seen by abnormal extensions in its caudal end, something which was not observed in WT samples (Fig. 1E,F).

### High GDNF levels result in short and misplaced ureters

**Figure 2:**
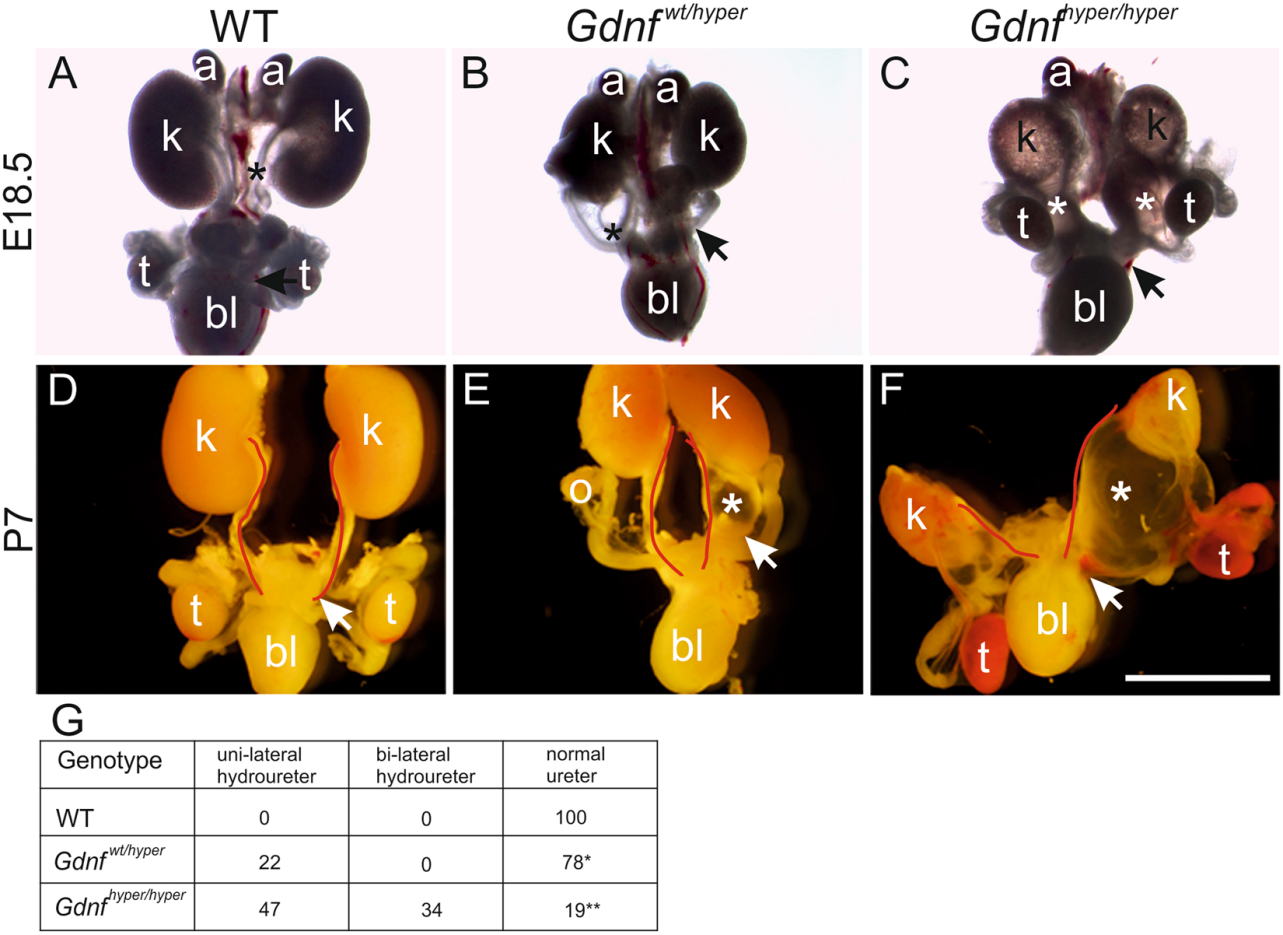
Kidney and lower urogenital tract defects in *Gdnf* hypermorphic mice. (**A**) Representative images of wild type (WT), (**B)** *Gdnf* ^*wt*/hyper^ and (**C)** *Gdnf* ^hyper/hyper^ urogenital system at E18.5 and (**D–F**) at P7, demonstrating renal hypoplasia, unilateral hydroureters (black & white asterisks) and severe renal hypodysplasia accompanied with short ureters. Red lines depict medial edge of ureter. Note hemorrhaging in testes. (**G)** Distribution of distinct ureter phenotype percentages in different genotypes at late embryonic and early postnatal stages (n = 38, 41, 32 for WT, *Gdnf* ^*wt*/hyper^ *Gdnf* ^hyper/hyper^, respectively). In the table, one asterisk indicates short ureter while two is severely shorter. Abbreviations: a; adrenal gland, bl; bladder k; kidney, o; ovary, t; testis. Scale bar: 250 µm.

The finding that the most caudal ureteric bud that gives rise to the future ureter fails to separate from the nephric duct in *Gdnf*  ^hyper/hyper^ embryos predicts potential defects in later development of the ureter and other nephric duct derivatives. Examination of the urogenital system at late-embryonic and early-postnatal stages indeed revealed unilateral hydroureters in *Gdnf*  ^wt/hyper^ kidneys, which were often also unilaterally hypoplastic and irregularly shaped (Fig. 2A,B,E,G). In *Gdnf*  ^hyper/hyper^ animals the entire urogenital system was severely malformed and kidneys were small and multicystic as previously described^26^ (Fig. 2A–F). Almost half of the *Gdnf*    ^hyper/hyper^ and 1/5 of the *Gdnf*   ^wt/hyper^ mice also develop hydroureters (Fig. 2G), which is a hallmark of abnormal ureter-to-bladder connection. Accordingly, ureteric bud and collecting duct cysts were regularly seen in postnatal kidneys (Fig. S3C–E)^26^. Strikingly, the ureter length in *Gdnf*   ^hyper/hyper^ kidneys at E18.5 (56.6 ± 12.9%, n = 3) was markedly shorter than that seen in WT controls (100 ± 7.7% n = 4, p = 0.028), and therefore kidneys were postnatally positioned low in the abdominal cavity (Fig. S3A,B, see also Fig. 2C,F). This indicates that GDNF regulates ureter length and thereby indirectly the final localization of the kidneys in the adult body.

### Abnormally wide primary ureteric bud in *Gdnf*^hyper/hyper^ embryos associates with a proliferation burst in the early nephric duct

**Figure 3:**
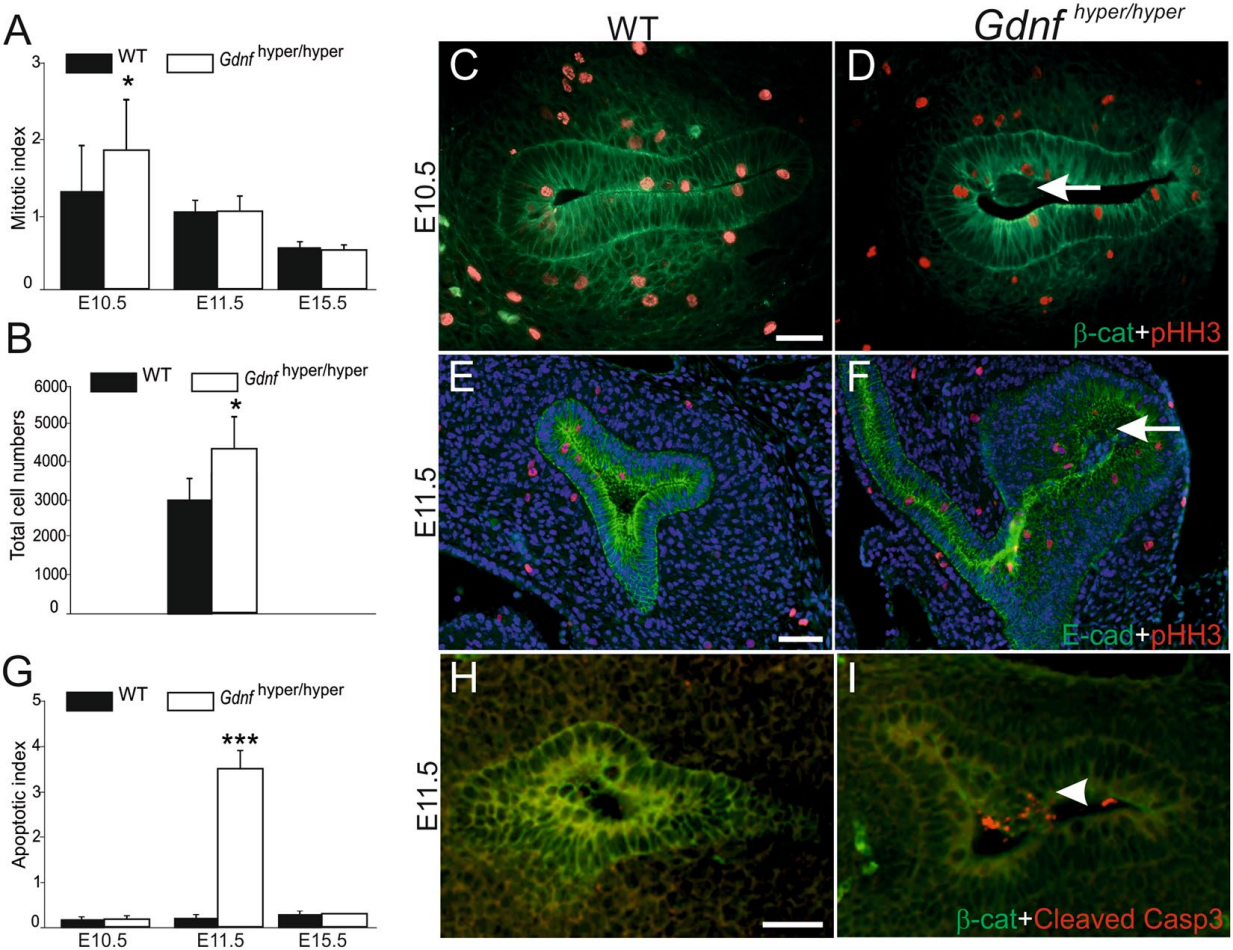
GNDF augments mitosis in caudal nephric duct at the time of ureteric bud outgrowth. (**A**) Mitotic indices were determined as the percentage of pHH3-positive cells within b-catenin or E-cadherin positive epithelium. Graphs show indices of wild type (WT, black bars) and *Gdnf* ^hyper/hyper^ embryos (white bars) in caudal nephric duct (E10.5), ureteric bud epithelium (E11.5) and ureteric bud tips (E15.5). A statistically significant increase in mitotic indices was observed at E10.5 samples (7–9 vibratome sections per embryo, n = 3/genotype, p < 0.05, Student’s t-test). (**B**) Total cell counts in E11.5 WT and *Gdnf* ^hyper/hyper^ ureteric bud tips (p < 0.05). (**C**) Representative E10.5 WT and (**D**) *Gdnf* ^hyper/hyper^ kidney primordia shown after staining with pHH3 (red) and b-catenin (green). (**E)** E11.5 WT kidney shows typical T-shaped ureteric bud (E-cadherin, green), but (**F**) *Gdnf* ^hyper/hyper^ epithelium is abnormally enlarged without normal branching pattern. Arrows in (**D**) and (**F**) point to cell mass accumulated in the luminal side of the epithelium. (**G**) Regardless of the genotype, labeling for cleaved Caspase3 does not reveal apoptosis in the kidney primordia of E10.5 embryos (n = 7) while a statistically significant increase is seen at E11.5 in *Gdnf* ^hyper/hyper^ epithelium (white bar, n = 5 for WT and 4 for *Gdnf* ^hyper/hyper^, Student’s t-test, p < 0.001). At E15.5 cleaved caspase 3 labeling reveals similar amount of apoptotic cells in the cortex of WT (n = 3) and *Gdnf* ^hyper/hyper^ (n = 2) kidneys. (**H**) An example of WT kidney stained with cleaved Caspase3 (red, n = 5) shows virtually no apoptosis, while (**I**) numerous apoptotic cells (arrowhead) are detected in the ureteric bud lumen of *Gdnf* ^hyper/hyper^ embryos at E11.5 (n = 4). Scale bars 50 µm. Error bars on all graphs represent standard deviation.

Next we sought to characterize the cellular causes for the abnormalities in primary UB and distal ureter morphogenesis leading to short and misconnected ureter in *Gdnf* ^hyper/hyper^ animals. We first examined proliferation and apoptosis in control and hypermorphic kidneys and found that at the time of primary ureteric bud formation (E10.5), the mitotic index in the caudal ND and ureteric epithelium of *Gdnf* ^hyper/hyper^ was 40% higher than in the controls (Fig. 3A). This excess proliferation at the time of primary UB formation likely explains the observed increase in primary ureteric bud size in early *Gdnf* ^hyper/hyper^ kidneys (Fig. 1). Surprisingly, the mitotic index was normalized in tips of *Gdnf* ^hyper/hyper^ UBs soon after the onset of branching, despite the significant difference in the total ureteric epithelial cell numbers (Fig. 3A,B). Interestingly, accumulation of cells negative for mitosis marker pHH3 were observed in the luminal cavity of UB both at E10.5 and E11.5 in *Gdnf* ^hyper/hyper^ kidneys, while these were not seen in WT kidneys (Fig. 3C–F). Simultaneously, apoptosis was significantly increased in *Gdnf* ^hyper/hyper^ ureteric bud epithelium (Fig. 3G–I). This data show that excess GDNF increases cell proliferation in the caudal nephric duct, specifically at the emergence of the ureteric bud, and suggest that later in UB epithelium excess GDNF may interfere with luminal mitosis mechanism^27^ as accumulation of cell mass and increased apoptosis were observed in the lumen of *Gdnf* ^hyper/hyper^ UB.

Previously, it has been indicated that apoptosis is an essential process in common nephric duct remodelling^18,28,29^. We stained common nephric ducts with apoptosis marker cleaved Caspase3 and found reduced apoptosis in subset of *Gdnf* ^hyper/hyper^ samples at E11.5 representing the stage for the onset of remodelling (Fig. S4A,B). Despite the clear defect in nephric duct morphology at later stages, the amount of apoptotic cells in all *Gdnf* ^hyper/hyper^ samples was comparable to WT NDs at at E12.5 (Fig. S4C,D). Staining of the lower urinary tract samples at E14.5 showed that timing of apoptosis was not changed in *Gdnf* ^hyper/hyper^ samples as again they were comparable to WTs (Fig. S4E,F). This suggests that common nephric duct remodelling utilizes additional mechanisms than apoptosis to achieve normal morphological outcome.

### High GDNF levels block ureteric bud and renal growth by restricting emigration of progenitors from ureteric bud tips

**Figure 4:**
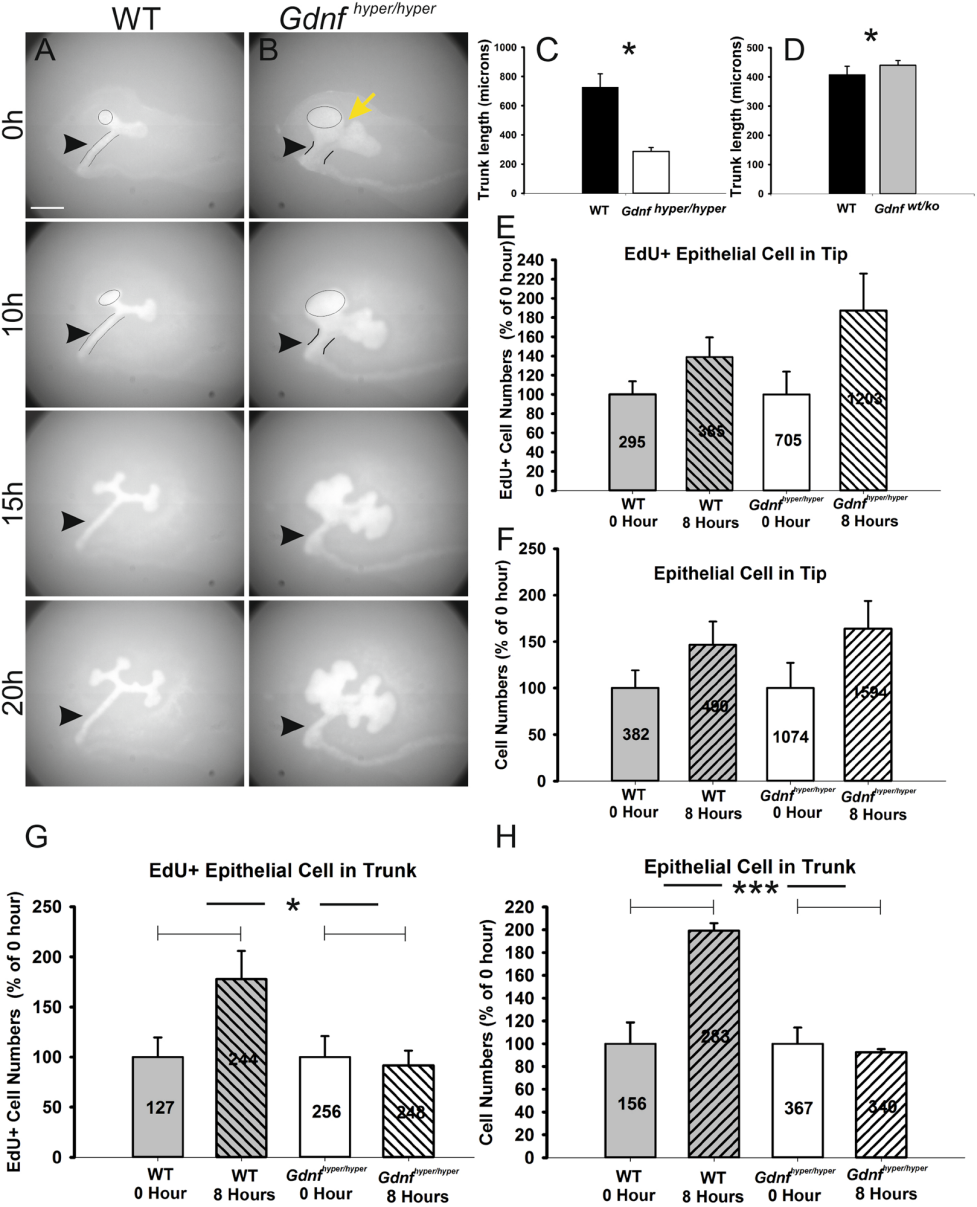
*In vitro* analysis of ureteric bud and trunk growth. Time-lapse imaging of 24 h cultured E11.5 (**A**) wild type (WT) and (**B**) *Gdnf* ^hyper/hyper^ kidneys where nephric ducts and ureteric bud epithelia are visualized by transgenic Hoxb7CreGFP expression. Yellow arrow marks cleft-like furrow between the distinct ends of ureteric bud tips, arrowhead points to ureteric trunk. (**C**) Measurement of trunk lengths, presented as the average primary trunk length ± SEM, in WT and *Gdnf* ^hyper/hyper^ kidneys at E12 reveals that UB trunks are significantly shorter in *Gdnf* ^hyper/hyper^ kidneys (728.6 ± 91.3 µm in WT, 286.5 ± 28.0 µm in *Gdnf* ^*hyper/hyper*^, n = 4/genotype, p = 0.013, unpaired two-tailed Student’s t-test). (**D**) Trunk measurements in WT and *Gdnf* ^*wt/ko*^ kidneys show that trunk length in *Gdnf* ^*wt/ko*^ kidneys is increased (408.9 ± 27.9 µm in WT (n = 13), 439.8 ± 16.0 µm in *Gdnf* ^wt/ko^ (n = 16), p = 0.019, unpaired two-tailed Student’s t-test). Analysis of (**E**) EdU-positive cell counts and (**F**) total epithelial cell counts (average cell numbers depicted inside the bars on (**E**–**H**) in ureteric bud tips (marked by black circles in A and B at 0 and 10 h images) are shown as percentage of growth rates (WT, n = 5; *Gdnf* ^hyper/hyper^, n = 4). Growth rate at 0 h was set to 100% in both genotypes. The increase ratio in wild type cell numbers was approximately the same in EdU+ (38.9 ± 20.5%, n = 5) and total cell counts (46.6 ± 25.2%, n = 4), while both EdU+ cells (87.5 ± 38.3%, n = 3) and total cells (63.9 ± 30%, n = 3) increased remarkably more in *Gdnf* ^hyper/hyper^ tips. (**G**) Corresponding analysis of growth rates in the ureter trunks of E11.5 WT and (**H**) *Gdnf* ^hyper/hyper^ kidneys. Dramatic increase in both EdU+ cells (77.9 ± 28.1%, n = 4) and total cell numbers (99.2 ± 6.6%, n = 3) were observed in WT kidney trunks. *Gdnf* ^hyper/hyper^ trunk cells failed to increase either EdU+ (91.6 ± 14.7%, n = 3) or total cell numbers (92.6 ± 2.6%, n = 3). The area of measurements is depicted in A and B images of 0 and10h as black lines. Scale bar: 200 µm.

To analyze the effect of excess GDNF on further UB morphogenesis we crossed *Gdnf* ^wt/hyper^ mice with HoxB7CreGFP mice expressing green fluorescent protein (GFP) in the ND and its derivatives^30^. Normally, new ureteric bud tips are generated from the existing tips, which first expand to form the ampulla that then bifurcate to generate two new tips^8,31^. Bifurcation is followed by trunk elongation and some cells initially found in the tips are later found in the trunk region where the collecting duct differentiation takes place^32^. Indeed, time-lapse imaging of control kidneys showed typical, long and elongated trunks between the nephric duct and tip epithelia (Fig. 4A, movie 1). Control kidneys produced new branches from the ampulla, and also showed obvious trunk elongation. The UB tips in *Gdnf* ^hyper/hyper^ kidneys, however, were enlarged and exhibited a cleft-like furrow in the middle and bud-like structures all over the tips (Fig. 4B, movie 2). The *Gdnf* ^hyper/hyper^ tip expanded considerably more than the trunk - the future ureter, elongated, and remained significantly shorter than in WT controls (arrowheads in Fig. 4A,B; trunk lengths at E12.0 kidneys exemplified in Fig. S5A,B: 1135.2 ± 284.4 µm in WT (n = 5), 446.3 ± 87.3 µm in *Gdnf* ^hyper/hyper^ (n = 5), p = 0.0018). Thus, the ureteric bud tip growth occurred at the expense of trunk elongation in *Gdnf* ^hyper/hyper^ embryos.

Next we investigated how tips in *Gdnf* ^hyper/hyper^ kidneys remain enlarged despite a normal mitotic index after primary budding and why trunks in *Gdnf* ^hyper/hyper^ kidneys fail to elongate at a similar pace as in the WT. Highly proliferative tips host progenitor cells, which are capable of populating the renal collecting duct system^32–35^. Normal RET and its downstream activities appear critical for maintaining epithelial progenitor cells in the tip domain^32,36^. On the other hand, cells with low or no RET activity are left behind from the tip domain (the niche) and are later found in trunks where they differentiate into collecting duct cells. We hypothesized that a high GDNF level positively regulates progenitor expansion and has a negative effect on trunk elongation by restricting emigration of progenitors from the niche. If this is correct, low GDNF levels should result in longer trunks. The analysis of trunk lengths in *Gdnf*^wt/ko^ kidneys, which have only one functional allele expressing *Gdnf*^37^ (Fig. S5C,D), indeed revealed a small but statistical increase in trunk length (Fig. 4D, 408.93 ± 27.86 µm in WT (n = 13), 439.84 ± 16.02 µm in *Gdnf* ^wt/ko^ (n = 16), p = 0.019). This suggests that low GDNF dosage allows more cells to leave the tips to populate the trunks for elongation and differentiation.

Next, tip and trunk segments (Fig. S5E) were analyzed separately in *Gdnf* ^hyper/hyper^ kidneys. Previous characterization of cell cycle lengths in the developing kidney (14.5 h in tips and 22.4 h in trunks^35^) was utilized in designing the pulse-labelling experiments (5-ethynyl-2′-deoxyuridine (EdU), 30 min). Similar to mitotic index (Fig. 3A,B), EdU+ (295 ± 40 in WT (n = 5); 705 ± 168 in *Gdnf* ^hyper/hyper^ (n = 3)) and total cell (382 ± 73 in WT (n = 4); 1074 ± 293 in *Gdnf* ^hyper/hyper^ (n = 3)) counts were higher in *Gdnf* ^hyper/hyper^ tips than in WT tips (Fig. 4E,F). On average, 77% (75.59 ± 3.05%) of the WT (n = 4) tip cells and 66% (67.62 ± 3.63%) of the tip cells in *Gdnf* ^hyper/hyper^ kidneys (n = 3) were in S-phase (EdU+/total cells) at the initiation of the culture period (0 h). The proportion of EdU+ cells increased to 79% (78.78 ± 2.80%) in WT (n = 6) and to 75% (76.70 ± 5.79%) in *Gdnf* ^hyper/hyper^ tips at 8 h, showing a greater proportional increase with excess GDNF (Fig. S5F). This indicates that tip cells cycle faster in UBs of *Gdnf* ^hyper/hyper^ kidneys. This also suggests that shorter cell cycle time contributes to increased total tip cell numbers and ureteric bud size in *Gdnf* ^hyper/hyper^ kidneys.

Control ureteric bud trunks are longer than those in *Gdnf* ^hyper/hyper^ kidneys (Fig. 4A–C). Indeed, while EdU+ and total cell number almost doubled in the trunks of WT kidneys at 8 h, in sharp contrast neither parameter increased in the *Gdnf* ^hyper/hype^ trunks during this time period (Fig. 4G,H). These results imply that high GDNF levels result in large tips by the combination of two mechanisms: by restricting emigration from the tip niche to the trunks and by shortening the cell-cycle time in the tips. Alternatively, it is also possible that an excess of GDNF may have a direct negative effect on trunk cell proliferation, but this is unlikely as ureteric trunk cells do not express any known GDNF receptors^13^.

### MEK inhibition normalizes ureteric bud morphology and branching in *Gdnf*^hyper/hyper^ kidneys

**Figure 5:**
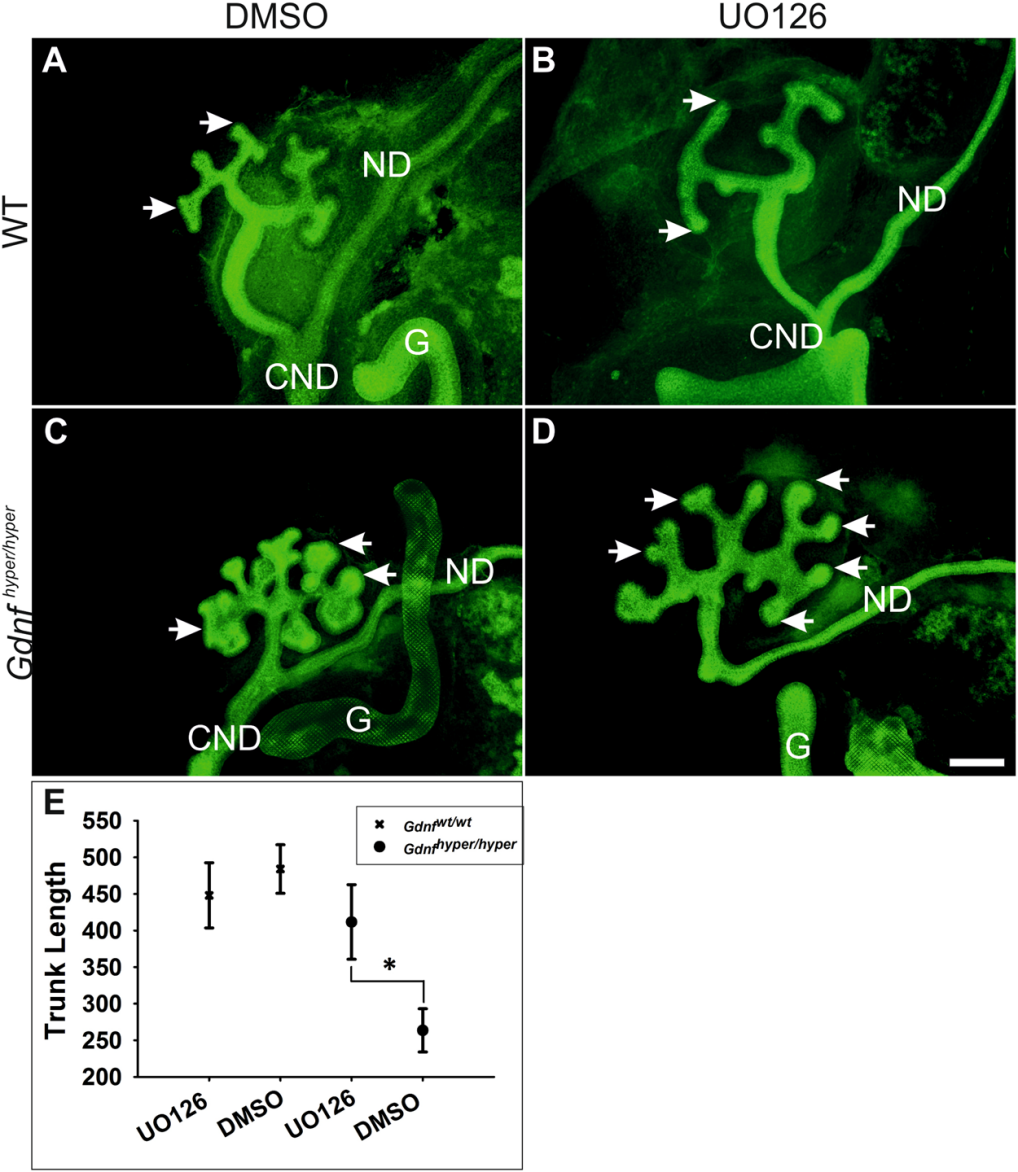
MEK inhibition rescues ureteric bud morphology in *Gdnf* ^hyper/hyper^ kidneys. (**A**) Wild type (WT) kidney cultured in mock medium for 48 h shows normal morphology of ureteric bud tips (arrows). (**B**) Similarly to published results^39,41^, inactivation of MAPK pathway by MEK inhibitor UO126 results in failure to expand ureteric bud tips (arrow) in WT kidneys. (**C**) *Gdnf* ^hyper/hyper^ kidney cultured in mock medium shows expanded ureteric bud tips and very short trunks. (**D**) MAPK pathway inhibition significantly improves both ureteric bud tip morphology and trunk length in *Gdnf* ^hyper/hyper^ kidneys. (**E**) Statistical analysis of UO126 treatment effects on trunk lengths in WT and *Gdnf* ^hyper/hyper^ kidneys shows that MEK inhibition significantly promotes trunk length in *Gdnf* ^hyper/hyper^ kidneys (411.69 ± 50.93 µm in *Gdnf* ^hyper/hyper^ kidneys cultured with UO126, 263.59 ± 29.55 µm in *Gdnf* ^hyper/hyper^ kidney cultured with DMSO, p = 0.047, n = 9, paired sample t-test; 448.01 ± 44.49 µm in WT kidney cultured with UO126, 484.03 ± 33.16 µm in WT kidney cultured with DMSO, p = 0.146, n = 3, paired sample t-test). Data presented as average trunk length ± the standard error of mean (SEM), *denotes P < 0.05 in paired sample t-test. Abbreviations: CND; common nephric duct, G; gut, ND; nephric duct. Scale bar: 200 µm.

Several intracellular cascades are activated downstream of RET receptor upon GDNF binding^38^. In order to assess which of these intracellular cascades could meditate GDNF’s effect on collecting ductal progenitors and ureter lenght we next performed chemical inhibition experiments in cultured kidney explants to examine potential predominant intracellular pathway in *Gdnf* ^hyper/hyper^ kidneys. Consistent with previous reports, inhibitor-specific responses were observed in WT kidneys where PI3K/AKT or MAPK cascades were inhibited^39–41^. Interestingly, only MAPK pathway suppression with MEK inhibitor UO126 normalized UB tip morphology and improved trunk length in *Gdnf* ^hyper/hyper^ kidneys (Fig. 5). Chemical MEK inhibition, similarly to its genetic abrogation specifically in UB and derivatives, results in elongation only phenotype, where UB tips fail to expand into an ampullae and rarely produce new tips^41^ (Fig. 5A,B). The UB tips in *Gdnf* ^hyper/hyper^ kidneys are wide and expanded but showed significant normalization upon 48 h culture with MEK inhibitor (Fig. 5C–E). Our results thus indicate that a MAPK dependent imbalance in collecting ductal progenitor self-renewal vs. differentiation drives the ureteric bud phenotype in *Gdnf* ^hyper/hyper^ kidneys. It remais to be seen whether MAPK pathway is responsible for mediating GDNF functions also in distal ureter remodeling.

### Endogenous overexpression of GDNF causes infertility in both genders

The “Ureteral Bud Theory” of Mackie and Stephens predicts that changes in the location where primary UB forms affect further ureter development^10^. Accordingly, rostrally expanded budding site in *Gdnf* ^hyper/hyper^ embryos results in failure to segregate the ureter from the ND ureter (Figs 1 and S2). In addition to giving rise to ureteric bud, segments of nephric duct also differentiate into the vas deferens and epididymal ducts in males, while in females nephric duct guides Müllerian duct development^9,11^. We hypothesized that defects in ND and distal ureter remodelling may lead to impaired development of reproductive organs whose differentiation also depends on nephric duct. Indeed, the hypothesis is supported by the finding that *Gdnf* ^hyper/hyper^ mice showed occasionally attachment of the ureter to the testis (Fig. 2F).

*Gdnf* ^hyper/hyper^ mice survive less than three weeks^26^ and therefore we could not test their fertility potential. However, we noticed that *Gdnf* ^wt/hyper^ mice became infertile after back-crossing  original mixed C57Bl/6NCrl/129SvOla background strain to the isogenic C57Bl/6NCrl strain. As an example, 9/22 *Gdnf* ^wt/hyper^ males in F1 generation and all *Gdnf* ^wt/hyper^ males in F2 (n = 9) failed to impregnate WT females despite clear vaginal plugs. Likewise, 10/22 *Gdnf* ^wt/hyper^ females in F1, and all *Gdnf* ^wt/hyper^ females in F2 were infertile, setting a limit on the number of possible backcrosses. As a consequence, the *Gdnf* ^wt/hyper^ mice had to be maintained in mixed background (C57BL/6NCrl/ICR/and129SvOla) to avoid infertility and produce the mouse colony for the experiments.

**Figure 6:**
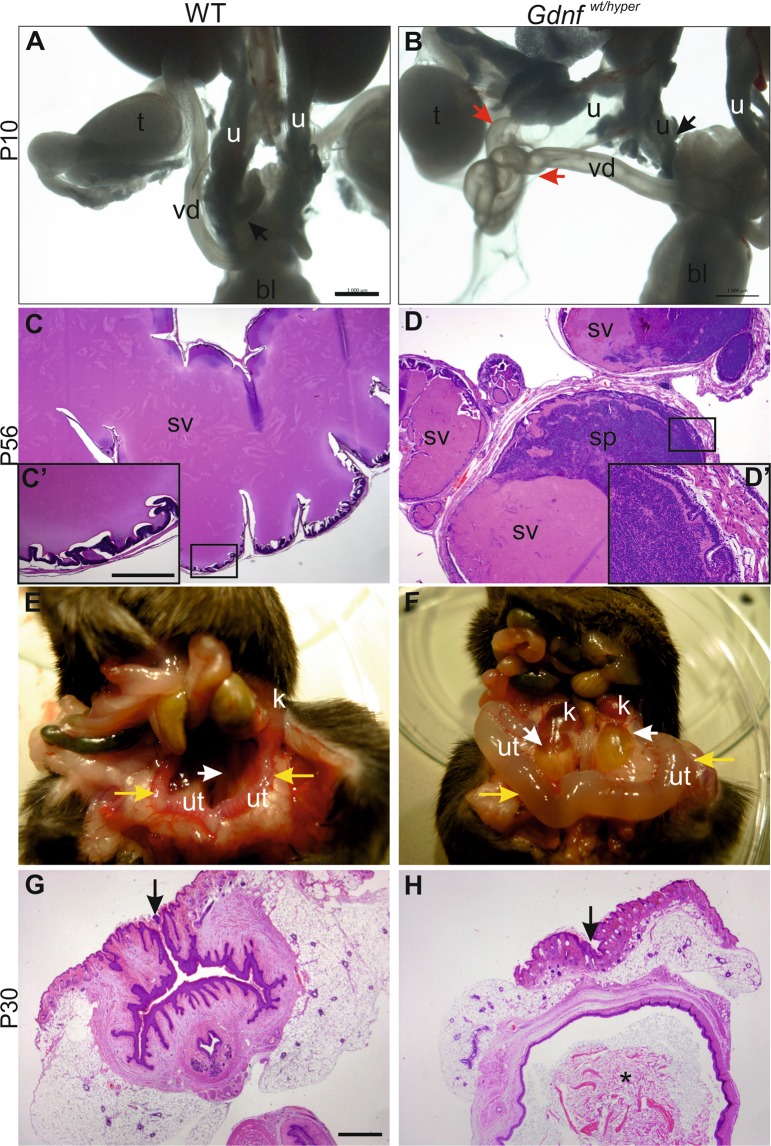
Reproductive organ defects in *Gdnf* ^wt/hyper^ mice. (**A**) Male wild type (WT) lower urinary tract and reproductive organs at P10. Black arrow denotes ureter (u) connection to bladder (bl). (**B**) *Gdnf* ^wt/hyper^ male in F2 generation exhibits split ureter with one (u, black arrow) connecting to bladder (bl) and the other, hydroureter (u) connecting to vas deferens (vd, red arrow). (**C**) Hematoxylin-eosin staining of P56 wild type seminal vesicle. Squared area is enlarged in C’. (**D**) Histology of P56 *Gdnf* ^wt/hyper^ seminal vesicle shows sperm inside the lumen and loss of typical lobular shape of seminal vesicles. Squared area is enlarged in D’. (**E**) Normal female mouse abdomen at P35. Yellow arrows point to uterus, white arrows indicate ureter. (**F**) Corresponding abdomen of *Gdnf* ^wt/hyper^ female shows hydrotic ureters and badly swollen uteri (yellow arrows). Hematoxylin-eosin staining of frontal sections of (**G**) WT and (**H**) *Gdnf* ^wt/hyper^ vagina at the surface level. Asterisk in H shows accumulation of keratinized mass inside the vagina, which is indicative of fluid blockage. Black arrows point to vaginal opening in wild type mice, and to the site where vaginal opening should be in *Gdnf* ^wt/hyper^ mice. All images are examples of F2 generation of backcrossing to isogenic C57BL/6NCrl background. Abbreviations: bl; bladder, sp; sperm, sv; seminal vesicle, t; testis, u; ureter, ut; uterus, vd; vas deferens. Scale bars: 1 mm and C’: 250 µm.

We next sought to understand the causes of infertility in sub-isogenic *Gdnf* ^wt/hyper^ mice. Anatomical examination of the F1 and F2 *Gdnf* ^wt/hyper^ males in the C57BL/6NCrl backcross revealed defects in the connections between the ureter, vas deferens, and seminal vesicles (Fig. 6A,B). For example, vas deferens was directly connected to seminal vesicles, which caused abnormal accumulation of sperm in the seminal vesicles of *Gdnf* ^wt/hyper^ male mice (Fig. 6C-D’). However, sperm collected from the epididymis appeared normal both in morphology and motility (Fig. S6A,B). Infertility in females was likely caused by an imperforate vagina (Figs 6E–H, S6C,D), which prevents penetrance of the sperm to the female oviduct. These data show that enhanced GDNF levels cause defects not only in ureter maturation but have also functional consequences on fertility through the control of reproductive organ development in both sexes.

## Discussion

The functional importance of the 3′UTR on executing gene function is currently unknown for most genes. Very little is known about how the kidneys are positioned in the abdominal cavity and how ureter length is specified. Here we report that GDNF levels, as regulated by its functional 3′UTR, define ureter length and thereby delineate the position of the kidneys in abdominal cavity. Furthermore, we find that correct GDNF levels are important for normal reproductive organ development in both genders.

Our results also highlight that genetic studies on 3′UTRs can provide information, which complements and enhances existing knowledge from gene deletion or mutational analysis. Gene deletion studies have shown that GDNF is required for the initiation of kidney development, but no defects in reproductive organ development has been reported^8,20,22,42–44^. Furthermore, knowledge on endogenous GDNF’s function beyond the induction of ureteric bud outgrowth and bifurcate branching is largely lacking. We found that GDNF regulates the expansion of collecting ductal progenitors, a cell population located in the ureteric bud tips capable of populating the entire collecting duct system^35^. Specifically, we revealed that an enhanced GDNF level increases the number of collecting ductal progenitors at the expense of the elongation of the trunk, the future ureter, thereby resulting in short ureters. Our results suggest that GDNF may participate in regulation of a novel form of cell divisions called luminal mitosis. It was shown that dividing cells of ureteric bud delaminate from the epithelial sheet to enter the luminal space of the epithelium^27^. This occurs just prior the cell division, after which the two cells re-enter the epithelial sheet couple of cells apart. Our observation of abnormal cellular mass in the lumen of *Gdnf* ^hyper/hyper^ UB tips together with accelerated cell cycle and increased apoptosis implies defects in re-entry process of luminal mitosis, but further experiments are needed to verify this hypothesis.

We also found that excess GDNF can result in infertility in adults likely due to defects in common nephric duct remodeling, a developmental process necessary for segregation of ureters from reproductive organs and known to depend on GDNF receptor RET^12,17,18^. The precise mechanism how GDNF regulates female reproductive tract development and specifically vaginal perforation remains to be determined. Previously GDNF was shown to induce proliferation in the urogenital sinus^45^, which functions as a precursor for the bladder and lower urogenital system (prostatic and penile urethrae in males; urethra and lower vagina in females). Changes in this process may contribute to development-derived infertility in *Gdnf* ^hyper/hyper^ mice and make an important aspect for future studies.

Interestingly, activating and inactivating mutations reported in Gdnf receptor RET do not phenocopy urogenital system malformations we found in *Gdnf* ^hyper/hyper^ mice. Activating RET mutations, which cause constitutively active signaling, result in pediatric cancer syndromes such as multiple endocrine neoplasia (MEN) type 2A and 2B^46,47^ but except for the rare occasions (total of five patients in three distinct kindreds^48–50^ of MEN2A), the majority of MEN patients display normal kidney morphology. Indeed, while urogenital system development in MEN2B mice is normal^47^, we found that *Gdnf* ^hyper^ mice display multiple defects not only in kidney but also in reproductive organs. Further, manipulation of specific docking sites responsible for activation of distinct intracellular cascades downstream of RET have resulted in varying degrees of changes in morphology of the kidney, nephric duct and its derivatives^8,12,17,18,51^. This, together with our results, suggests that a precisely tuned signaling cascade, where regulation of expression levels of the ligand, GDNF, is essential for defining the activation amplitude and cell-type specific responses in GDNF-RET regulated tissues.

Finally, our finding that increased GDNF levels due to disruption of its 3′UTR function - not mutations in GDNF or in its receptor protein encoding sequences^20,22,52,53^ - result in phenotype homologous to human CAKUT and infertility, may be clinically informative because despite of the advances in sequencing techniques, the disease causing mutation remains unknown in most patients suffering from CAKUT and infertility^2,54^. It is becoming increasingly clear that mutations in coding sequence of DNA alone do not account for many human disorders and epigenetic as well as other mechanisms controlling gene expression levels may explain many congenital diseases.

## Methods

Mouse models, genotyping and ethics statement. Generation of *Gdnf* ^hyper/hyper^ and knockout mouse lines used in the study was described by Kumar *et al*.^26^. Both lines were maintained on triple mixed background (C57BL/6NCrl/ICR/and129SvOla) for all experiments except those examining reproductive organs (indicated in the text). Hoxb7CreGFP^30^ line was bred to *Gdnf* ^hyper/hyper^ mice to enable visualization of ND and its derivatives. The genotyping was performed by PCR as described previously^20,26^.

Embryonic staging was determined as reported previously^55^ and all experiments were approved by the Finnish National Animal Care and Use Committee. The experiments were designed according 3R principles and followed European Union directives (Directive 2010/EU/63) in compliance with the Code of Ethical Conduct for Animal Experimentation.

### Tissue collection, processing, hematoxylin-eosin (HE) and immunofluorescent staining

Embryos and tissues of indicated stages were dissected in *Dulbecco*’*s* medium supplemented with 0.2% bovine serum albumin and fixed with 4% PFA. Further processing for paraffin embedding was done according to standard procedure with an automatic tissue processor (Leica ASP 200).

HE, whole-mount, and immunostaining on paraffin sections were performed as previously described^55^. More details in supporting information file.

### Organ culture, live imaging and EdU-labelling

Kidneys were cultured on a Trowell-type system in medium of F12:DMEM/10% fetal bovine serum/Glutamax/penicillin-streptomycin, and either processed for whole-mount immunofluorescent staining (see above) or imaged with an epifluorescent microscope (see below). The rudimentary lower urogenital blocks were dissected from *Gdnf* ^hyper/hyper^ and Hoxb7CreGFP; *Gdnf* ^hyper/hyper^ embryos at E10-11.5 and halved along the linea mediana ventralis before placing for culture on permeable support (Transwell®; Costar).

The kidneys used for ureteric bud trunk length measurements were either cultured for 1 h and followed by whole-mount immunofluorescent staining (n = 5 for WT and *Gdnf* ^hyper/hyper^ at E12, and n = 11 in WT and n = 7 in *Gdnf* ^wt/ko^ at E11.5), or measured directly from stereomicroscopy images of E18.5 kidneys (n = 4 in WT, n = 3 in *Gdnf* ^hyper/hyper^) by using ImageJ software. See the details of live imaging with time-lapse microscopy and EdU pulse labelling experiments in supplemental information. Statistical analyses were performed with unpaired two-tailed Student’s t-test or paired-samples t-test, when appropriate. The level of statistical significance was set to p < 0.05. The calculations were carried out with SPSS (IBM; Version 22).

### Quantification of mitotic indices and apoptosis

Quantification of mitotic index at indicated stages was performed on vibratome sections (three embryos per genotype and 7–9 sections per kidneys were studied for pHH3+ nephric duct/ureteric bud cells) similarly as previously described^56^. Proliferation significances were tested with Independent samples t-test (1-tailed, equal variances).

Apoptosis was analyzed on vibratome sections by double staining with cleaved-Caspase3 and β-catenin. Samples were collected at E10.5 (n = 7 for both genotypes), E11.5 (wt n = 5, *Gdnf* ^hyper/hyper^ n = 4) and E15.5 (wt n = 3, *Gdnf* ^hyper/hyper^ n = 2), and cleaved-Caspase3 positive cells were counted per section in each genotype for statistical testing with Independent samples t-test (1-tailed, equal variances).

### Sperm motility test

For motility testing, sperm from three F1 and two F2 *Gdnf* ^wt/hyper^ male mice backcrossed to C57BL/6NCrl isogenic background for one or two generations, respectively, were collected. Cauda was dissected out and two small incisions were made to allow sperm to swim out into HTF medium containing BSA (K-RVFE-50, William A. Cook Australia Pty. Ltd) for 15 min at 37 °C (N_2_/6% CO_2_/5% O_2_), after which sperm were counted according to standard categorization criteria (a-b-c-d) using a Bürker hemocytometer chamber (Hawksley). After motility assessment, a fraction of the sperm was pipetted onto a microscope slide for morphological analysis followed by HE staining.

## Supplementary information

Electronic supplental information

Movie 1: Wild type kidney branching

Movie 2: GDNF hypermorphic kidney branching.
