# Supplementary material for: Development of the urogenital system is regulated via the 3′UTR of GDNF
Source: Sci Rep. 2019 Mar 28;9:5302. doi: 10.1038/s41598-019-40457-1 (PMC6438985; doi:10.1038/s41598-019-40457-1)
Supplement: Supplementary file 1 — Electronic supplental information [file 41598_2019_40457_MOESM1_ESM.pdf]

Development of the urogenital system is regulated via the 3'UTR of GDNF

Li, Hao[1,7]., Jakobson, Madis[2,7]., Ola, Roxana[2,3]., Gui, Yujuan[1]., Kumar, Anmol[1]., Sipilä, Petra[4]., Sariola, Hannu[2]., Kuure, Satu[1,5, 8]. & Andressoo, Jaan-Olle[1,6,8].

[1] Institute of Biotechnology, Helsinki Institute of Life Sciences and Faculty of Medicine, [2] Institute of Biomedicine, Biochemistry and Developmental Biology, University of Helsinki, Finland,[3] Department of Basic, Preventive and Clinical Science, Faculty of Medicine, University of Transylvania, Brasov, Romania and Functional Genomics, Proteomics and Experimental Pathology Department, Prof. Dr. I. Chiricuta Oncology Institute, Cluj-Napoca, Romania [4] Research Centre for Integrative Physiology and Pharmacology, and Turku Center for Disease Modeling (TCDM), Institute of Biomedicine, University of Turku, Finland, [5] Laboratory Animal Centre, University of Helsinki, Finland, [6] Department of Neurobiology, Care Sciences and Society, Karolinska Institutet, Sweden

[3, 6, 7] Current addresses

[7]Co-first author, [8]Co-last author

Corresponding authors: satu.kuure@helsinki.fi
tel: +358 2941 59395,
cell: +358 40 770 6242,

tel: +358 2941 59394
cell: +358 50 358 1213

SUPPLEMENTARY INFORMATION

Extended Materials and Methods

Animals

All animal work was conducted according to European Union directives (Directive 2010/EU/63), as well as in compliance with the Code of Ethical Conduct for Animal Experimentation. The licenses for mouse lines used in this publication were KEK16-020 and ESAVI/11198/04.10.07/2014.

Mice were housed in individually ventilated cages with optimal humidification and heating and maintained in the certified animal facilities. Cleaning, disinfection and general care is provided by the professionals and following mandatory federal and internal regulations.

Tissue processing

For hematoxylin-eosin (HE) staining, the tissues were dissected and collected for overnight fixation with 4% PFA (pH 7.4), and further dehydrated and paraffinized with an automatic tissue processor (Leica ASP 200). The paraffin-embedded tissues were sectioned at 5 µm. Selected sections were dewaxed in Xylene followed by rehydration with a graded ethanol series (100-75-50-25%) before being stained with Harris hematoxylin and Eosin. Finally after dehydration, the sections were mounted with Coverquick 2000 (PROLABO®; VWR Chemicals).

Vibratome sections (10-50µm) were cut with microtome (HM650, Microm Int.) from PFA-fixed E13 urogenital blocks, which were embedded in 4% low melting agarose (NuSieve GTG, Lonza).

For whole-mount IHC, the tissues were first dissected and cultured followed by fixation and permeabilization with ice-cold 100% methanol for 10 min before the overnight incubation with primary antibodies. Next day the tissues were vigorously washed with PBST (0.1% Tween 20 in PBS) several times (5-8x 1h) after which the samples were further incubated with the relevant species-specific secondary antibodies overnight before imaging.

For immunofluorescence on sections, the tissues were dissected and collected for overnight fixation with 4% PFA (pH 7.4). The selected paraffin sections were gradually rehydrated through an ethanol series after deparaffinization followed by heat induced antigen retrieval in antigen retrieving buffer (20mM Tris, 1mM EDTA; pH 8.5). After

blocking with 10% fetal bovine serum (Hyclone) for 1 hour at room temperature, the sections were incubated with primary antibody overnight at 4 ℃ followed by incubation with the relevant species-specific secondary antibodies conjugated with Alexa Fluor® 488, Alexa Fluor® 594 or Alexa Fluor® 647 respectively, and stained for nuclei with Hoechst. The information of the primary and the secondary antibodies used is shown in **S1 table**.

Whole mount live imaging, trunk measurements and cell counting

Urogenital blocks were dissected at E11.5 for half an hour culture after which they were stained with anti-E-Cadherin antibody and anti-Sox9 antibody. All samples were imaged with 10X objective with epiluorescence microscope (Carl Zeiss) equipped with the HXP120 LED unit (Zeiss) and a high-resolution charge-coupled device camera (AxioCam HRc, Zeiss).

The measurement of primary trunk length was carried out in cultured E11.5 and intact postnatal kidneys using Zen lite software (2012 blue edition) through tracing the primary trunk manually with hand-held computer mouse (see Fig. S5A-E). Data is expressed as the average primary trunk length ± SEM and with sample size of four for wild type control and *Gdnf* $^{hyper/hyper}$, and eleven for wild type control and seven for *Gdnf* $^{wt/ko}$.

For live-imaging, kidney rudiments were cultured in the humidified chamber of a Marianas 3i (3I intelligent Imaging Innovations) set-up equipped with Zeiss Axio Observer Z1 fully motorized inverted microscope and motorized xy-Stage (ASI MS-2000) for a total of 24h. Images were taken every 30min and were processed for videos with Zeiss Zen software.

EdU pulse labelling was performed by applying a final concentration of 10µM EdU to the culture medium at the beginning of the culture period. After a 30min pulse, one half of the lower urogenital block of each embryo was collected for immediate ice-cold methanol fixation. The EdU-containing culture medium was replaced with normal medium for the other half of the lower urogenital block that was cultured for an additional 8 hours without EdU. Before fixation, the tissues were washed with PBS and EdU incorporation detection was performed with Click-iT EdU Alexa Fluor Imaging Kit (Molecular Probes/Invitrogen) according the manufacturer’s instructions.

Imaging for EdU and total cell count analysis was performed with LSM 700 confocal microscope (Carl Zeiss) equipped with the HXP120C LED unit (Zeiss) and a high-resolution charge-coupled device camera (AxioCam HRc, Zeiss). The kidneys were imaged with 20 X objective and Zen software (2012 SP1; black edition; 8.1.0.484) was utilized for image processing. Quantification of the number of epithelial cells was carried out by using Imaris software (version 7.2) through counting the nuclear Hoechst signal for total cell counts, and EdU signal for determining the number of epithelial cells in S/M phase of the cell cycle. The analysis was done at two distinct regions of ureteric bud epithelium; the tip region and ureter trunk region depicted in figures 4A-B at 0 and 10h time points.

Data is presented as the average number of total or Edu+ epithelial cells in the tips and trunks at given time point. For the graphs shown in Fig. 4, cell numbers at 0h as set to 100% and change in cell numbers presented as an increase or decrease to that. The graphs shown in Fig. S5F demonstrate the percentage of EdU+ cells among the total cell numbers at given time point in control and *Gdnf* $^{hyper/hyper}$ ureteric bud tips.

Imaging

Epifluorescent imaging was performed with a Zeiss Axio Imager M2 microscope equipped with a AxioCam HRc camera and Leica DM6000 microscope equipped with a HAMAMTSU Flash 4.0 camera. The images were processed with Zen software and LAS AF software, respectively. The imaging of whole-mount tissues was performed with a SteREO Lumar.V12 stereomicroscope equipped with AxioCam MRm camera (Carl Zeiss, Germany).

Confocal imaging was performed with Zeiss LSM 700 confocal microscope (Carl Zeiss, Germany) equipped with Zen software. The number of EdU-labelled ureteric bud epithelial cells and the total number of ureteric bud epithelial cells were counted from confocal images with Imaris software (Version 8.1; Bitplane).

***In vivo* fertility**

The breeding performance of nine F1 and six F2 *Gdnf* $^{wt/hyper}$ male mice in isogenic background was followed in normal mating conditions. Two-month-old males were mated with wt C57Bl/6N female mice, and the number of litters and offspring of each male was recorded during a time period of six months. The capability to mate was

tested by overnight breeding followed by examination of vaginal plug appearance in females mated with four F1 and three F2 *Gdnf* $^{wt/hyper}$ male mice in isogenic background. Values were compared to those obtained from *Gdnf* $^{wt/hyper}$ in mixed background male mice in breeding at the same time and in the same animal room.

S1 table .

Primary antibody	Host	Dilution	Supplier
mouse Epithelial cadherin	goat pAb	1:500/ 1:300	R&D Systems
cleaved-Caspase 3	rabbit mAB	1:200	Cell Signaling Technology
Sox9	rabbit	1:500	Millipore

Secondary antibody	Host	Dilution	Supplier
anti goat Alexa Fluor 488	donkey	1:400	Jackson Immuno Research Laboratories
anti rabbit Alexa Fluor 594	donkey	1:400	Jackson Immuno Research Laboratories
anti mouse Alexa Fluor 647	donkey	1:400	Jackson Immuno Research Laboratories

Supplementary Information Figures

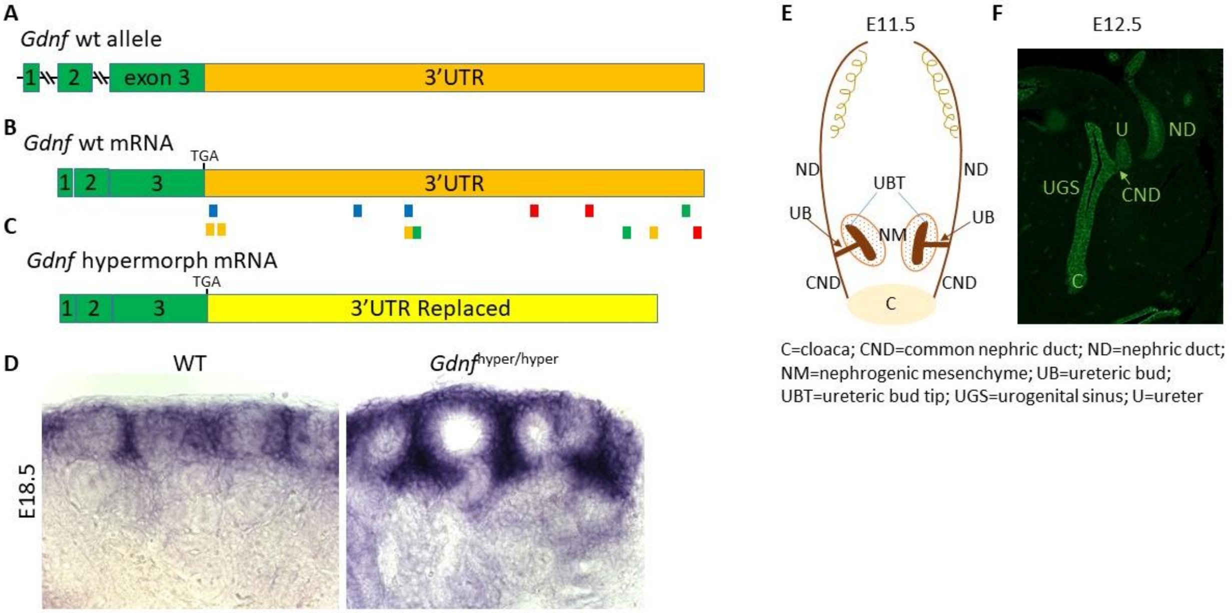

Figure 1. *Gdnf* targeting and early tissues in urogenital system development. A) *Gdnf* wt allele, B) the resulting mRNA, and C) *Gdnf* mRNA in GDNF hypermorph mice. The native *Gdnf* 3'UTR was replaced with a puro-tk cassette, which lacks microRNA (colored bars on B) and regulatory protein binding sites. D) *In situ* hybridization analysis of *Gdnf* expression patterns in wild type and *Gdnf*$^{hyper/hyper}$ kidneys at E18.5 shows similar localization of mRNA transcripts, which are remarkably enriched in *Gdnf*$^{hyper/hyper}$ kidneys. E)Schematic illustration of E11.5 urogenital system. Nephric ducts, which have grown down and made connection with cloaca, give rise to ureteric buds at the initiation of renal differentiation. Ureteric bud is subdivided into tip (UBT) – trunk (brown arrows) domains. Ureteric bud tips are surrounded by nephrogenic mesenchyme, which hosts a pool of nephron progenitors capable of differentiating into all segments of mature nephrons. F) E-cadherin immunofluorescent staining at E12.5 shows the geographical positioning of future ureter and common nephric duct in relationship with urogenital sinus, which is a specific epithelial segment in cloaca.

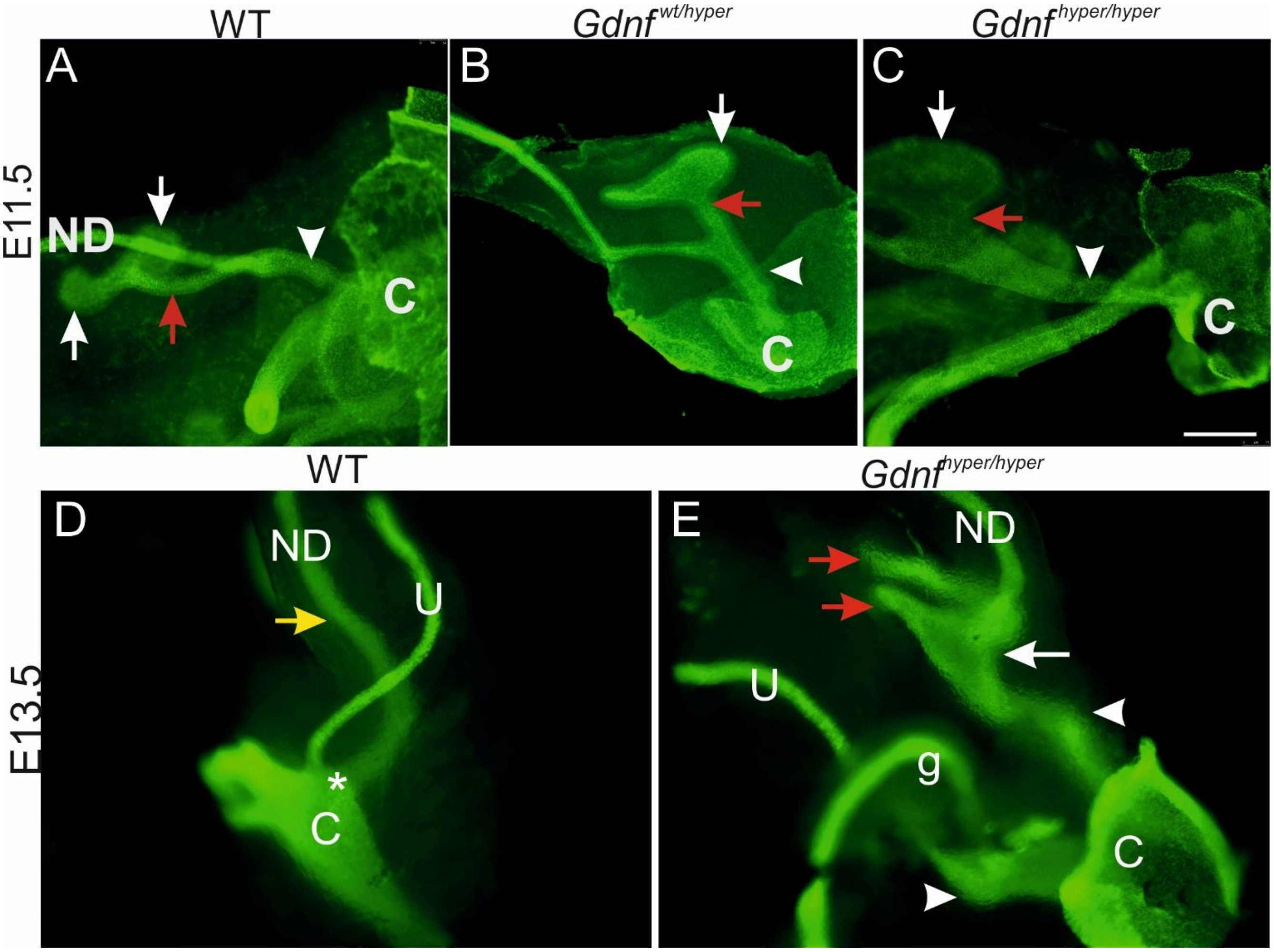

Figure 2. A) Wild type urogenital system at E11.5 shows clear separation of ureter (marked with red arrow) and nephric duct (ND), while connection to cloaca takes place through a segment of common nephric duct (arrowhead). B) Ureteric bud trunk is formed in *Gdnf* $^{wt/hyper}$ but it is shorter than in WT. C) *Gdnf* $^{hyper/hyper}$ has no distinct ureter trunk, but kidney (red arrow) is connected to cloaca via long and thick common nephric duct (arrowhead). D) Image of half of lower urogenital block of E13.5 wild type (WT) embryo showing proper separation of distal ureter from nephric duct (ND, yellow arrow). E) Entire lower urogenital block of *Gdnf* $^{hyper/hyper}$ embryo showing thick common nephric ducts (arrowheads), where one side (lower) shows distinction of distal ureter from nephric duct but at abnormally posterior position. The other side has extra buds (red arrows) connecting to the widened junction (arrow) between ureters, nephric duct and common nephric duct (arrowhead). Abbreviations: c; cloaca, g; gut, ND; nephric duct, u; ureter. Scale bar: A-C 200µm, D-E: 100µm.

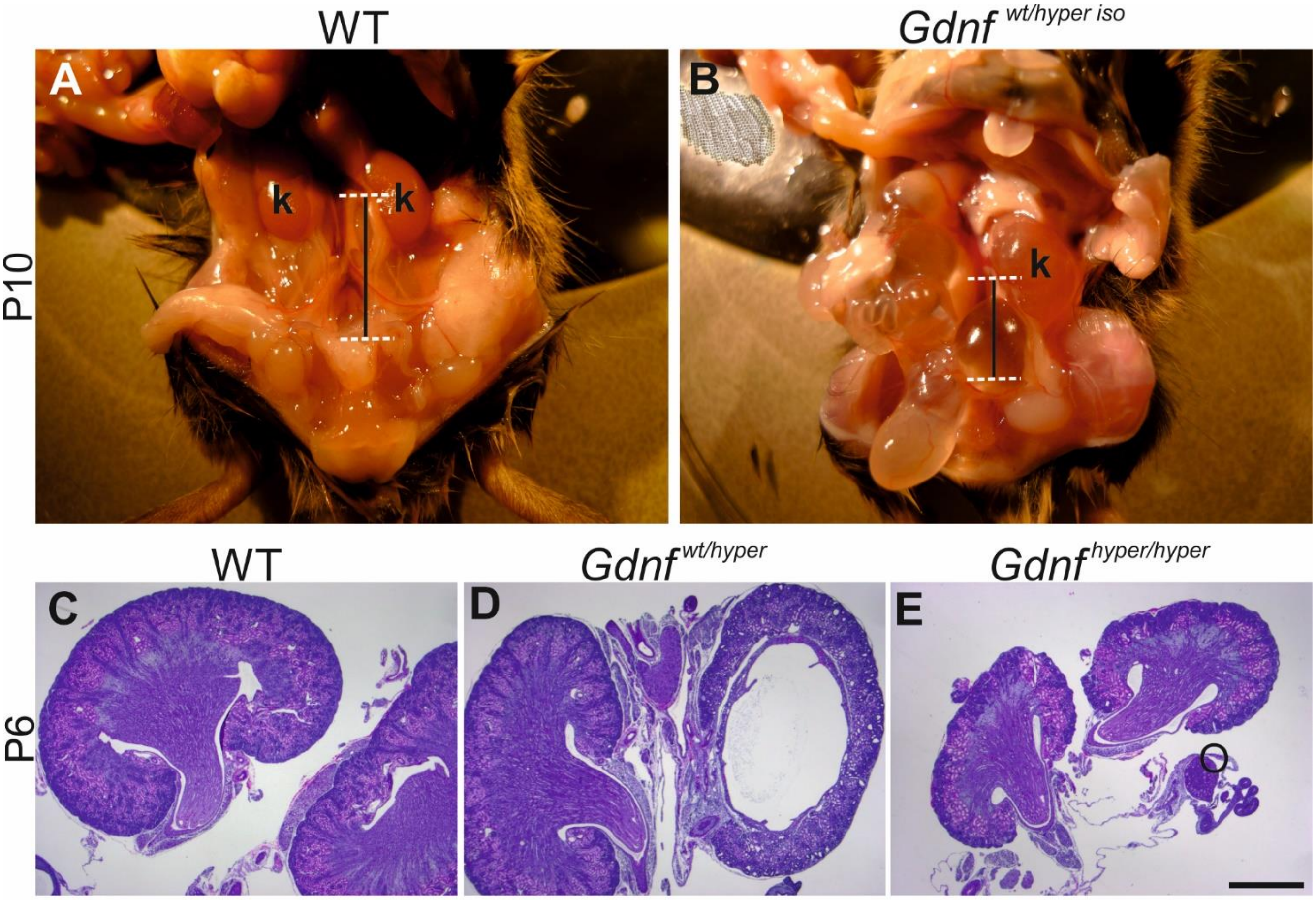

Figure 3. Representative images of mouse abdomen at P10 in A) wild type and B) *Gdnf* $^{\text{wt/hyper}}$ of F1 generation from backcrossing to C57/BL. White dotted lines connected with black line show the position of kidney relative to bladder. Histology of postnatal day 6 (P6) C) wild type, D) *Gdnf*$^{\text{wt/hyper}}$ and E) *Gdnf*$^{\text{hyper/hyper}}$ kidneys. Abbreviation: K; kidney, O; ovary. Scale bar 1mm.

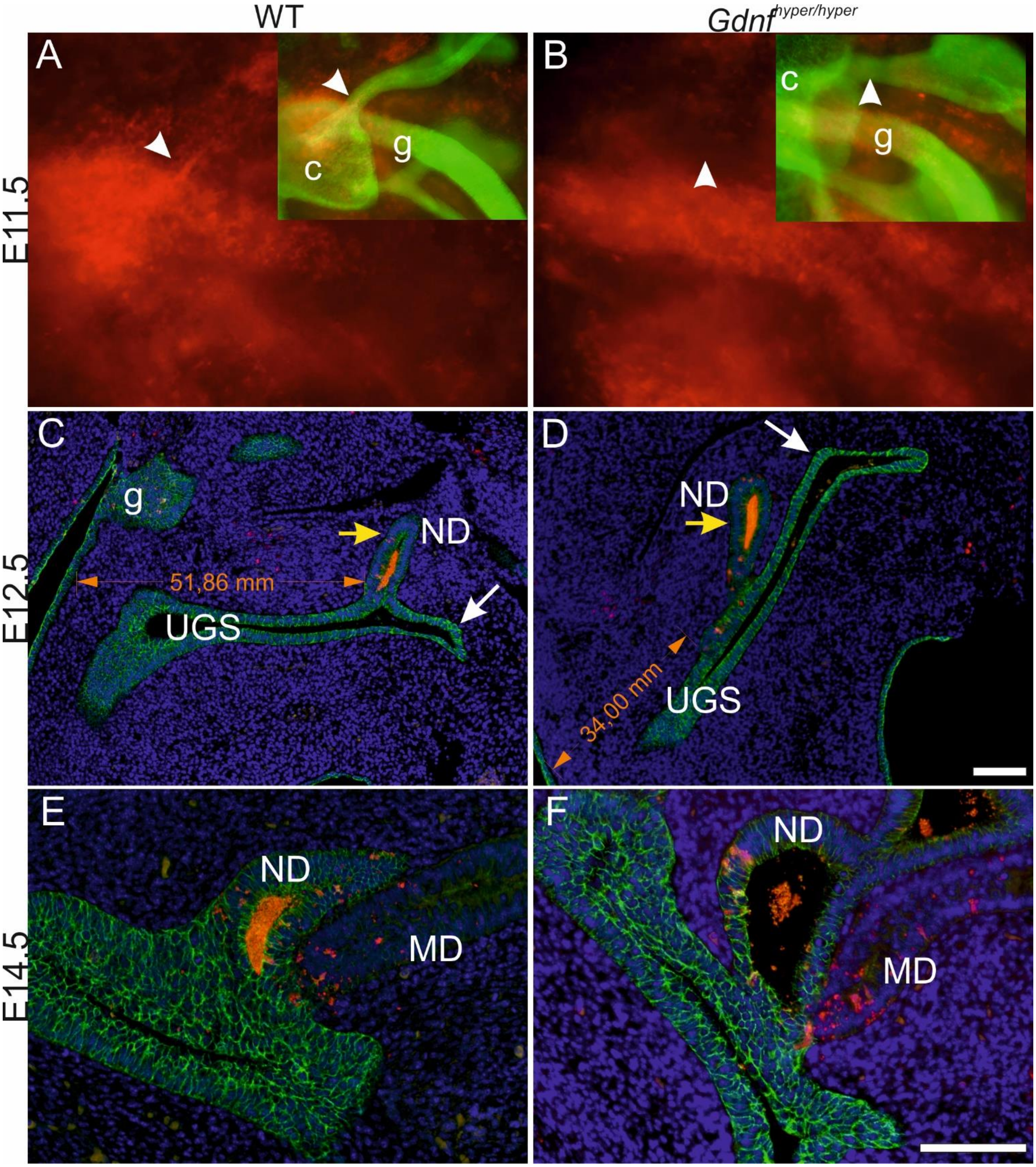

Figure 4. Whole mount staining of cleaved-Caspase3 (red) in E11.5 A) WT and B) *Gdnf* hyper/hyper urogenital system. Inserts show also E-cadherin (green) visualization of epithelial structures in developing urogenital system. Arrowhead points to the region in WT common nephric duct, which shows clear signal for apoptosis. Cleaved-

Caspase3 signal is markedly reduced in two out of seven *Gdnf* $^{hyper/hyper}$ common nephric ducts (arrowhead in B). C) E12.5 WT lower urogenital tract sections stained with E-cadherin (green) and cleaved-Caspase3 (red) show abundant apoptosis in nephric duct (ND, yellow arrow). D) Corresponding *Gdnf* $^{hyper/hyper}$ sections shows similar abundant apoptosis in nephric duct but also failure of nephric duct to connect to correct site at close proximity of primitive bladder (arrow). Instead, nephric duct connects to the segment of urogenital sinus (UGS) that is notably closer to cloaca membrane visualized by E-cadherin (orange 2-headed arrow). The distances between cloaca membrane and nephric duct connection to UGS are shown in orange, 2-headed arrow. E) Apoptosis (red, cleaved-Caspase3) in WT E14.5 nephric and Müllerian ducts is comparable to that detected in F) *Gdnf* $^{hyper/hyper}$ ducts, where nephric duct is abnormally enlarged. Abbreviations: c; cloaca; g, gut; MD, Müllerian duct; ND, nephric duct; U, ureter; UGS, urogenital sinus. Scale bar: 100µm.

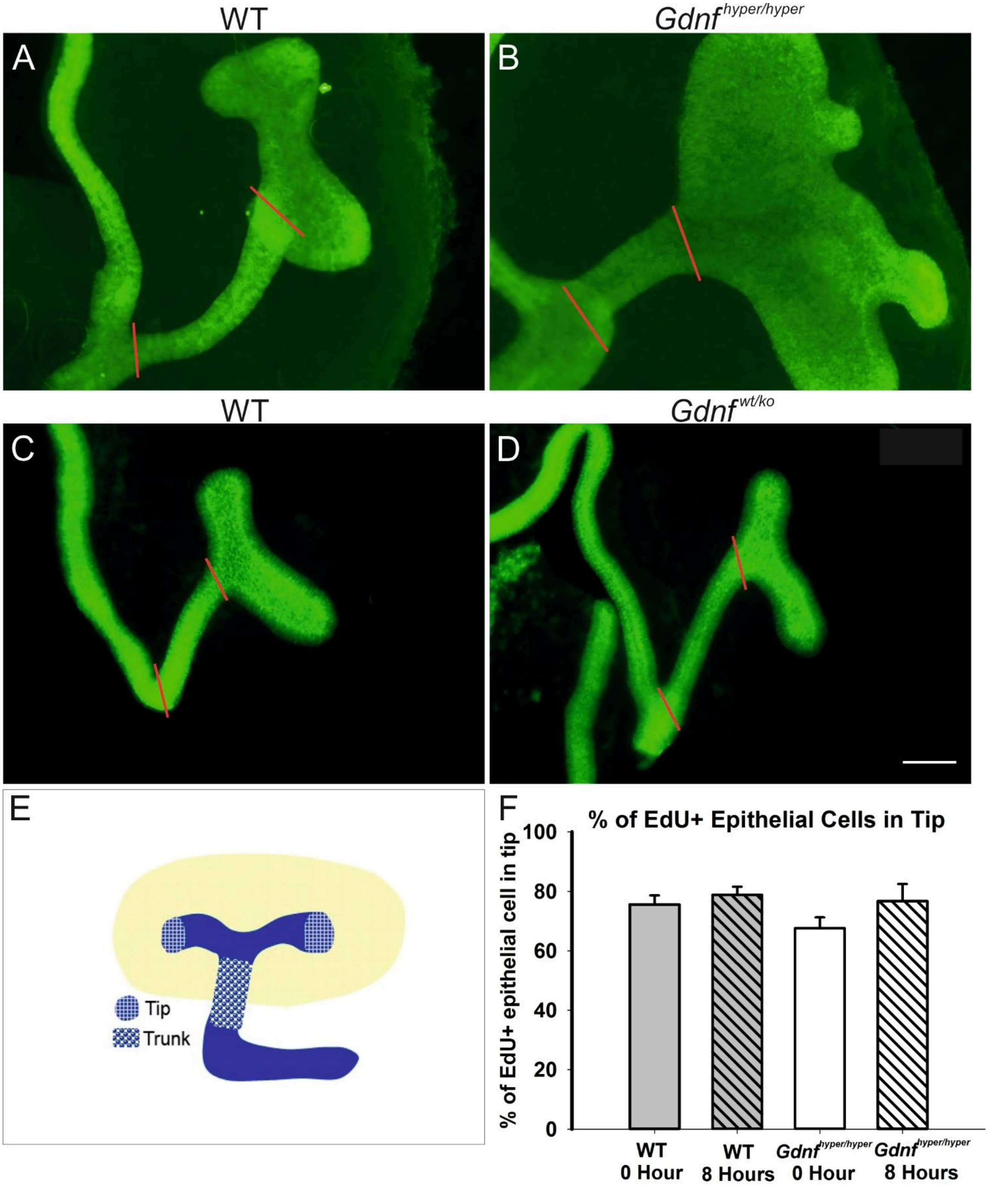

Figure 5. Examples of E12 whole mount A) wild type (WT) and B) *Gdnf* $^{hyper/hyper}$ kidneys stained with E-cadherin (green). C) WT and D) *Gdnf* $^{wt/ko}$ stained with E-cadherin (green). The trunk lengths were measured from the

depicted region between two red lines with Zeiss Zen lite software (2012 blue edition). E) Schematic demonstration of tip and trunk segments that were determined for Imaris program analysis of EdU+ and total cell numbers in Fig. 4E-H. F) Percentages of EdU+ cells out of total epithelial cells in wild type (WT) and *Gdnf* $^{hyper/hyper}$ ureteric bud tips at 0 and 8h time points. Scale bar 100µm.

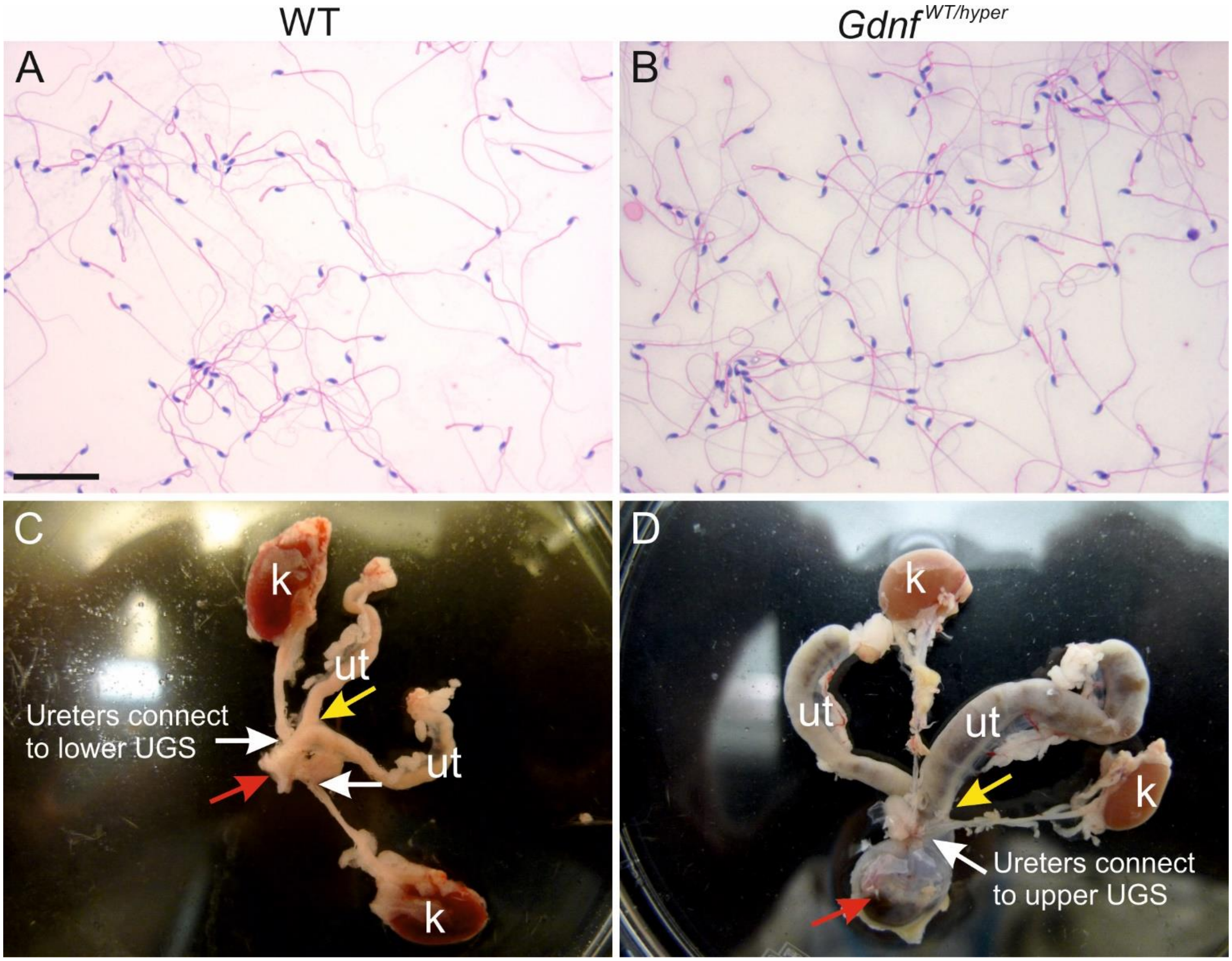

Figure 6. Hematoxylin-eosin staining of sperm collected from A) wild type (WT) and B) *Gdnf* $^{wt/hyper}$ epididymis from males in F1 back-crossing to C57BL/6NCrl strain. Evaluation of sperm motility in a swim test was performed prior to morphological analysis. C) F1 WT and D) *Gdnf* $^{wt/hyper}$ female urogenital systems at postnatal day 40. White arrows show connections to urogenital sinus (UGS), which are more posterior in control mice than in heterozygotes. Red arrow indicates vaginal opening, which is closed in 45% of *Gdnf* $^{wt/hyper}$ females in F1 generation. Yellow arrow points to the uterus-UGS connection site, which appears unchanged in all *Gdnf* $^{wt/hyper}$ females, although the uterine horns are severely swollen. Abbreviations: k, kidney; ut, uterus. Scale bar: A-B 50µm.
